# Near-infrared probes for luminescence lifetime imaging

**DOI:** 10.7150/ntno.63124

**Published:** 2022-01-01

**Authors:** Benhao Li, Jing Lin, Peng Huang, Xiaoyuan Chen

**Affiliations:** 1 Marshall Laboratory of Biomedical Engineering, International Cancer Center, Laboratory of Evolutionary Theranostics (LET), School of Biomedical Engineering, Shenzhen University Health Science Center, Shenzhen 518060, China; 2 Departments of Diagnostic Radiology, Surgery, Chemical and Biomolecular Engineering, and Biomedical Engineering, Yong Loo Lin School of Medicine and Faculty of Engineering, National University of Singapore, Singapore, 119074, Singapore; 3 Clinical Imaging Research Centre, Centre for Translational Medicine, Yong Loo Lin School of Medicine, National University of Singapore, Singapore 117599, Singapore; 4 Nanomedicine Translational Research Program, NUS Center for Nanomedicine, Yong Loo Lin School of Medicine, National University of Singapore, Singapore 117597, Singapore; In memory of Dr. Sanjiv Sam Gambhir and Dr. Moritz Kircher.

**Keywords:** Near-infrared probes, luminescence imaging, lifetime imaging, stimuli responsive, second near-infrared window

## Abstract

Biomedical luminescence imaging in the near-infrared (NIR, 700-1700 nm) region has shown great potential in visualizing biological processes and pathological conditions at cellular and animal levels, owing to the reduced tissue absorption and scattering compared to light in the visible (400-700 nm) region. To overcome the background interference and signal attenuation during intensity-based luminescence imaging, lifetime imaging has demonstrated a reliable imaging modality complementary to intensity measurement. Several selective or environment-responsive probes have been successfully developed for luminescence lifetime imaging and multiplex detection. This review summarizes recent advances in the application of luminescence lifetime imaging at cellular and animal levels in NIR-I and NIR-II regions. Finally, the challenges and further directions of luminescence lifetime imaging are also discussed.

## Introduction

Biomedical luminescence imaging has broad applications in life science due to its ability to achieve real-time investigation of physiological and pathological processes with fast feedback, high sensitivity, high spatiotemporal resolution 1-6. In order to act as luminescence contrast agents, a lot of luminescence probes, including organic dyes 7-20, quantum dots (QDs) 21-25, rare-earth doped nanoparticles (RENPs) 26-31, single-walled carbon nanotubes (SWCNTs) 32,33, metal-ligand complexes 34,35 and semiconducting polymer nanomaterials (SPN) 36,37 have been explored. Over the past few years, significant efforts have been made to create luminescence contrast agents that operate in the near-infrared window (700-1700 nm), which offers the enhanced tissue penetration over 5 mm 38-42. Especially, luminescence imaging in the second near-infrared window (NIR-II, 1000-1700 nm), which is also called shortwave infrared region (SWIR), has shown higher resolution at deeper tissue penetration compared with NIR-I (700-1000 nm) window due to the reduced tissue scattering and diminished autofluorescence 43-54. Meanwhile, recent works demonstrate that the autofluorescence of tissue becomes negligible for bioimaging beyond 1500 nm 55,56. Unfortunately, only a few probes can achieve bioimaging beyond 1500 nm 57,58. As we know, accurate detection and bioimaging in deep tissue is still hampered by the fact that luminescence signal intensity exhibits unavoidable and inhomogeneous attenuation caused by tissue absorption and scattering 59-61. Therefore, *in vivo* imaging of deep tissue requires the design and development of alternative techniques which is not based on the signal intensity.

Luminescence lifetime imaging has emerged as an important technique to detect or image specific biomarkers or physiological processes in living systems 62-64. Luminescence lifetime is the reciprocal of rate constants of return routes from excited state to ground state 65. The lifetime imaging requires imaging instrument to receive photons emitted from probes. Thus, the lifetime probes with long wavelength excitation and emission are capable of luminescence lifetime imaging in deep biological tissues. As for all luminescence parameters, the luminescence lifetime is the most direct insight into the interaction of contrast agents with their biological environment 66. Lifetime imaging can report on physiological and pathological events that are difficult or impossible to detect through luminescence intensity imaging, because lifetime imaging is independent of contrast agent concentration and tissue penetration 66,67. Compared with luminescence intensity, the luminescence lifetime provides a stable measurement method which is less susceptible to artifacts arising from tissue absorption and scattering, excitation intensity variations, probes photobleaching, or unknown tissue penetration depth 68.

## Luminescence lifetime imaging techniques

**Figure 1:**
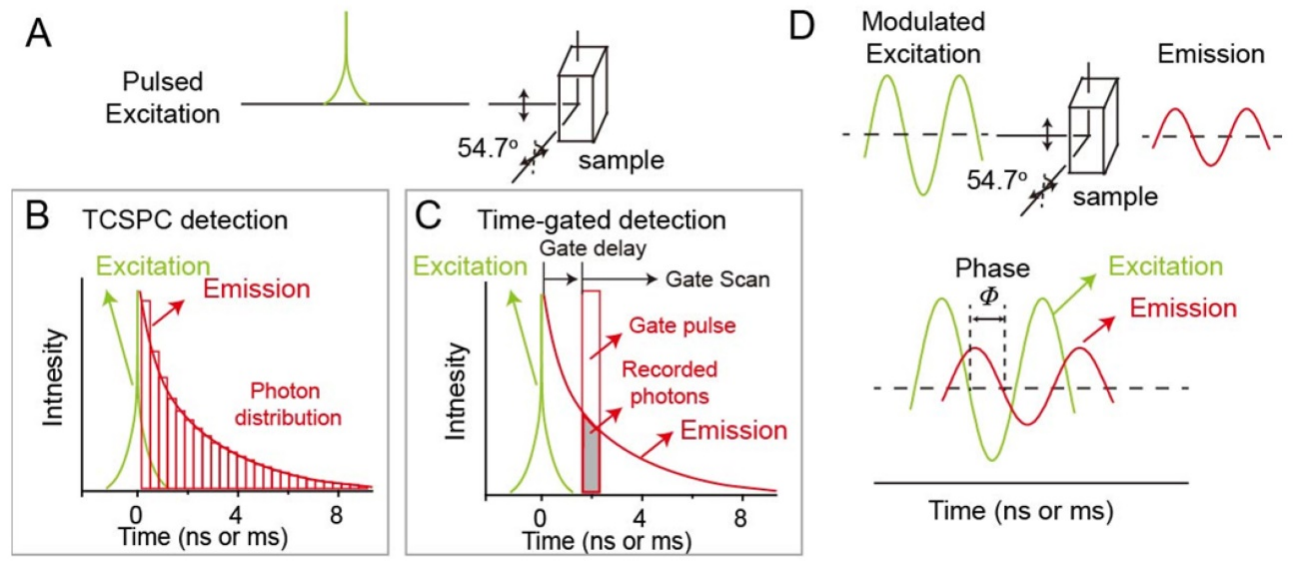
Luminescence lifetime imaging techniques. (A) Time-domain lifetime imaging techniques including time correlated single photon counting (TCSPC) detection (B) and time-gated detection (C). (D) Frequency-domain lifetime imaging technique.

Up to now, time-domain lifetime imaging techniques including time correlated single photon counting (TCSPC) and time-gated detections, and frequency-domain lifetime imaging technique, are two major ways to realize luminescence lifetime imaging. In time-domain fluorometry, the probes are excited by a pulsed light (**Figure 1A**). The width of pulsed light should be much shorter than the lifetime of the probes. After removing the excitation laser, the luminescence intensity of probes would decrease with the time. When the luminescence intensity decreases to the inverse of the natural base value e, the time duration required is called the luminescence lifetime τ. In order to avoid the influence of anisotropy and rotational diffusion on the intensity decay, in general, the intensity decays are measured by a polarizer at 54.7^o^ from the vertical z-axis. For now, within time-domain lifetime imaging techniques, TCSPC and time-gated detections are widely to achieve lifetime imaging. The principle of TCSPC detection is shown in **Figure 1B**. After pulsed light excitation, less than one photon is detected per laser pulse. Photons are detected and recorded in the histogram. The x-axis is the time difference between the time of detected photons and paused pulsed laser. The y-axis is the number of detected photons during the time difference. Therefore, the histogram represents the waveform of the decay. For time-gated detection, the decay functions are measured by consecutive scanning and imaging for different delay of the gate pulse (**Figure 1C**). In the frequency-domain lifetime imaging technique, the probes are excited under intensity-modulated light, typically sine-wave modulation (**Figure 1D**). Generally, the modulation frequency of excitation light will reach over 100 MHz. When the probes are excited, the emissions are compelled to follow the same modulation frequency. The lifetime of probes comes from the time delay of emission relative to excitation. The delay time is measured according to the phase shift (Φ), based on the following formula, τ_Φ =_ (1/ω) tanΦ, (ω: the modulation frequency in radians).

## Luminescence lifetime imaging applications in life sciences

Compared to intensity measurements, luminescence lifetime imaging with higher reliability is widely used in life sciences, such as protein interactions 69-71, biologically relevant ions 72,73, pH 74-76, temperature 77,78 *etc*. So far, luminescence lifetime imaging techniques have been used at the cellular level (luminescence lifetime imaging microscopy) 65,79-81 and at the animal level (wide-field luminescence lifetime imaging) 34,54,61. Meanwhile, luminescence lifetime imaging has been carried out from visible to NIR-I and NIR-II. In this review, luminescence lifetime imaging in NIR-I and NIR-II regions are summarized in Table 1.

### Luminescence lifetime imaging in NIR-I region

#### Non-activatable luminescence lifetime imaging

Luminescence probes which do not respond to stimuli in physiological conditions are called non-activatable luminescence lifetime probes. Non-activatable luminescence lifetime probes have robust imaging signal and are widely used for multiplexing. Multiplex detection and imaging have shown great potential in nucleic acid analysis, protein profiling, and clinical diagnosis, which can simultaneously detect and identify multiple molecular species in one sample with minimal volume 91-93. Optical multiplex imaging has become a promising method due to its advantages of flexibility, convenience, and fast feedback 94-96. However, conventional multiplex imaging based on luminescence emission wavelength and intensity are limited to spectral overlap and background interference. Luminescence lifetime of probes is independent of intensity and therefore more tolerant to the ambient background. Thus, luminescence probes with different luminescence lifetimes can locate different biomolecules more accurately. Meanwhile, this unique method has great potential for imaging fluorophores with similar excitation/emission wavelengths and different luminescence lifetimes. Therefore, the color/lifetime binary strategy could exponentially scale the encoding capacity, unlocking the limitation of the channel number during multiplexing.

**Figure 2:**
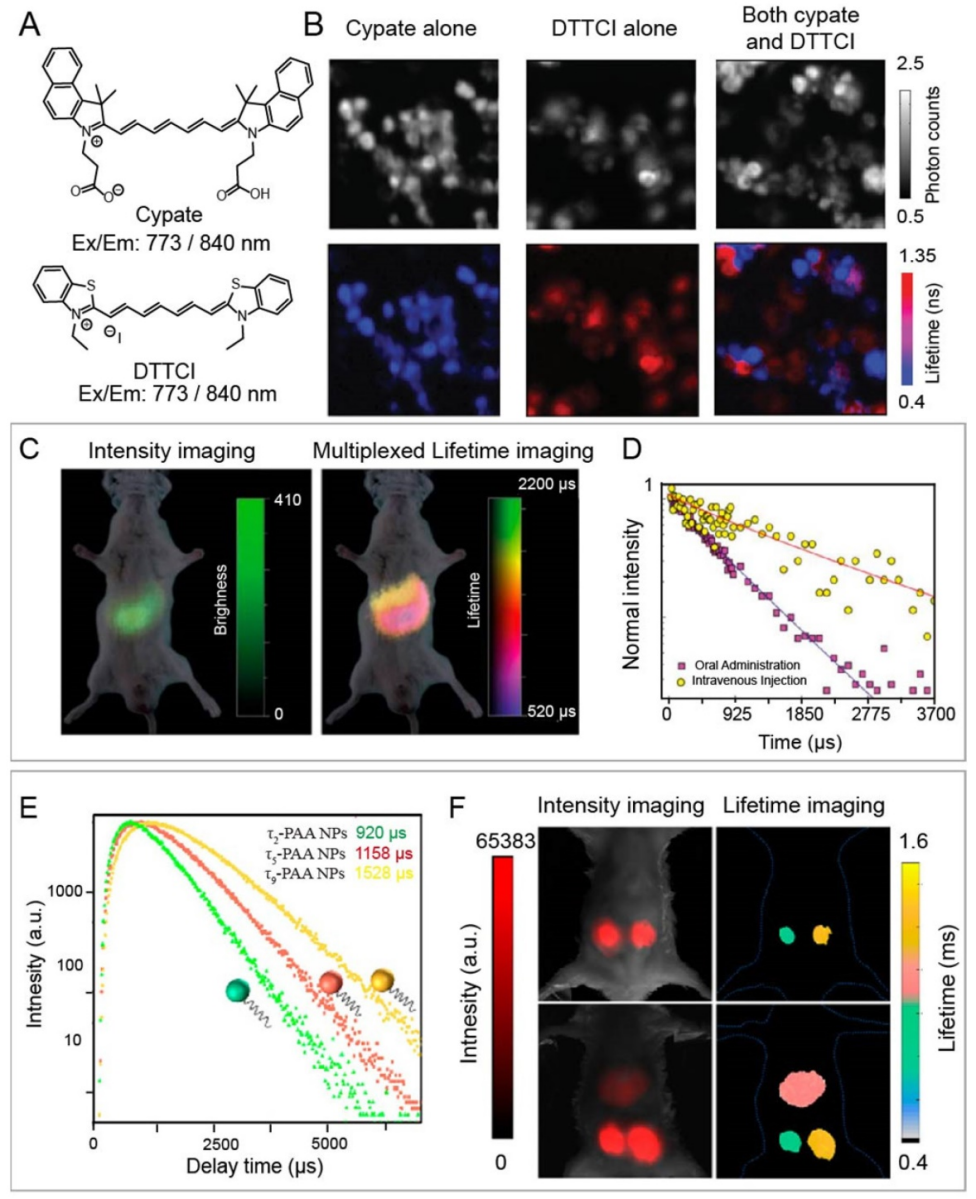
(A) Chemical structures of NIR-I dyes cypate and DTTCI. (B) luminescence intensity images (top row) and luminescence lifetime imaging microscopy images (bottom row) with cypate alone, with DTTCI alone, and with both cypate and DTTCI under 773 nm pulsed laser excitation. Adapted with permission from 82, copyright 2012. (C) Multiplexed intensity and lifetime images of healthy mice after oral and intravenous injection of Yb-doped RENPs. (D) Luminescence decay profile of the RENPs at two different locations. Adapted from permission from 83, copyright 2018. (E) Luminescence intensity decay of three poly(acrylic acid)-coated Tm-doped nanoparticles with different lifetimes of τ_2_, τ_5_, and τ_9_. (F) *In vivo* multiplexed lifetime imaging using three Tm-doped nanoparticles. Adapted with permission from 84, copyright 2020.

For example, Nothdurft and co-workers reported a home-made lifetime imaging system, including fiber coupled laser diode, a confocal laser scanning microscope, and TSCSPC card. This system can determine intracellular distributions of two NIR-I fluorescent dyes, including cypate and 3,3-diethylthiatricarbocyanine iodine (DTTCI), whose emission wavelengths are both located between 800 and 840 nm under 773 nm laser excitation (**Figure 2 A**,** B**) 82. Luminescence intensity failed to achieve multiplex imaging due to the similar excitation and emission wavelengths of cypate and DTTCI. However, co-incubated NIR-I dyes could be easily distinguished and located due to the different lifetime values of 0.5 and 1.1 ns through lifetime imaging. This approach provides a tool for monitoring the distribution of dyes with similar excitation wavelength at the cellular level. Unfortunately, this imaging system cannot be applied at the animal level. To solve this issue, Ortgies and coworkers have developed time-gated technique based lifetime imaging system for *in vivo* multiplexed NIR-II lifetime imaging at the animal level. A series of Yb^3+^-doped RENPs were constructed* via* dopant engineering method with different lifetime (0.1-1.5 ms). Luminescence lifetime imaging can distinguish the biological distribution of NaY_0.6_Yb_0.1_Nd_0.3_F_4_@CaF_2_ NPs and NaY_0.7_Yb_0.1_Nd_0.2_F_4_@CaF_2_ NPs with different lifetime times (0.7 and 1.3 ms) after oral and intravenous administration in the same emission channel **(Figure 2 C, D)** 83. Chen and coworkers developed Tm-doped upconversion nanoparticles with different lifetimes (from 78 to 2157 μs) and NIR-I luminescence at 808 nm under 980 nm laser **(Figure 2 E)**. High contrast lifetime imaging in the liver and two subcutis can be achieved *via* intravenous and subcutaneous administration of NPs **(Figure 2 F)** 84. Multiplexed lifetime imaging overcomes spectral overlap and background interference in wavelength/intensity-dependent multiplex imaging. Therefore, it is a promising imaging modality for monitoring and tracking the distribution of biomolecules labelled with dyes or nanoparticles with different lifetimes to understand physiological or pathological processes.

#### Activatable luminescence lifetime imaging

**Figure 3:**
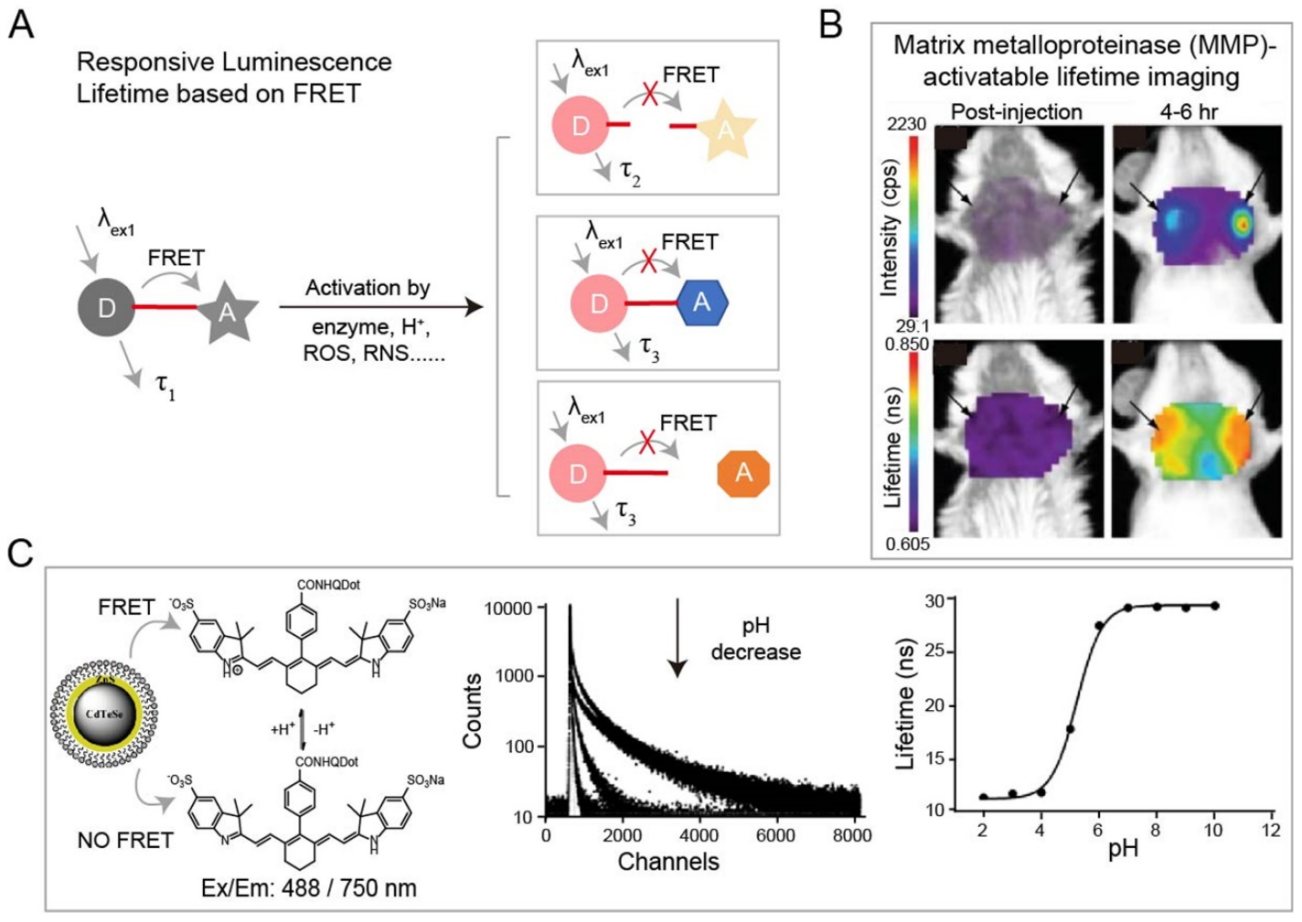
(A) Schematic of FRET-based luminescence probe and restoration of luminescence after stimulation by enzyme, H^+^, ROS, RNS etc. for lifetime imaging. (B) Luminescence imaging and luminescence lifetime imaging of breast tumors after injection of MMP750 (ex/em: 749/775 nm). Adapted with permission from 85, copyright 2011. (C) Illustration of FRET system of CdTeSe/ZnS quantum dots (QDs) and pH-sensitive organic dyes (left). Luminescence lifetime decay profile of the QD-dye system at different pH values (middle). Fit of the Luminescence lifetime vs pH (right). Adapted with permission from 74, copyright 2012.

The luminescence lifetime variation of probes responding to stimuli is the basis for a wide range of sensing applications. Förster resonance energy transfer (FRET) technique is the most common strategy for specific response to biological events both *in vitro* and *in vivo*. Adjacent dyes can interfere with the residence time of dyes in the excited state, resulting in a decrease of the mean residence time of dye in the excited state. In FRET system, luminescence lifetime of the energy donor decreases due to the energy transfer from energy donor to acceptor. After being activated by stimuli including H^+^, reactive Oxygen Species (ROS), reactive nitrogen species (RNS), lifetime recovery could be observed, which could be used for *in vitro* and *in vivo* detection (**Figure 3A**). This process will happen when the acceptor and donor are the identical dyes (homoFRET) or the different dyes (heteroFRET). When the dye is covalently attached to a polymer or biological macromolecules, the absorption and emission spectra will change, in most cases, the luminescence of dye will be quenched. Except the variation in luminescence intensity, the variation of lifetime can also be used to detect biological activity. NIR-I dyes, QDs and RENPs have been used to design FRET system with excellent luminescence lifetime biosensing. Solomon and coworkers reported a self-quenched matrix metalloproteinase (MMP)-activatable FRET probe using MMPSense750 (ex/em: 749/775 nm), which is used for a murine orthotopic breast cancer model through both luminescence intensity and lifetime imaging (**Figure 3B**) 85. This probe showed significant increases in luminescence intensity (10-fold) and lifetime (1.2-fold) at 4-6 h post-injection, suggesting the upregulated MMP activity in tumor tissue. Goergen and coworkers employed enzyme activatable probe, PGC-800 (ex/em: 778/794 nm), for imaging the expression of cathepsin B in mouse infarcted myocardium 86. Due to the cleavage of specific amide bonds by cathepsin B, the lifetime of probe recovered from 0.29 ± 0.01 to 0.47 ± 0.01 ns. Most importantly, luminescence lifetime imaging can distinguish the cathepsin B overexpressed infarct area from normal liver. Meanwhile, Alford and coworkers reported a self-quenched probe that is composed of antibody trastuzumab (targeting human epidermal growth factor receptor-2, HER2) conjugated to Alexa Fluor750 with different ratios of 1:8 or 1:1 (ex/em: 749/775 nm) for monitoring cellular internalization by luminescence intensity and lifetime imaging. These results proved that HER2 cells can be monitored using tumor-specific activatable lifetime probe 97. Achilefu and coworkers designed a pH sensing FRET nanomaterial of CdTeSe/ZnS QDs and LS662 (**Figure 3C**) 74. The nanomaterials transfer the pH sensitivity of LS662 to long-lifetime QDs, thereby inducing a reversible luminescence lifetime variation between 29 ns at pH >7 and 12 ns at pH <5. Recently, Li and coworkers reported a FRET nanocomposite consisting of Tm^3+^-doped RENPs (as donor) and IR-820 (as acceptor) for luminescence lifetime imaging 87. In the presence of ClO^-^, IR-820 would be destroyed, leading to luminescence lifetime recovery of RENPs from 200 µs to 600 µs at 800 nm. The ClO^-^ responsive luminescence lifetime imaging was successfully performed in a mouse arthritis model.

**Figure 4:**
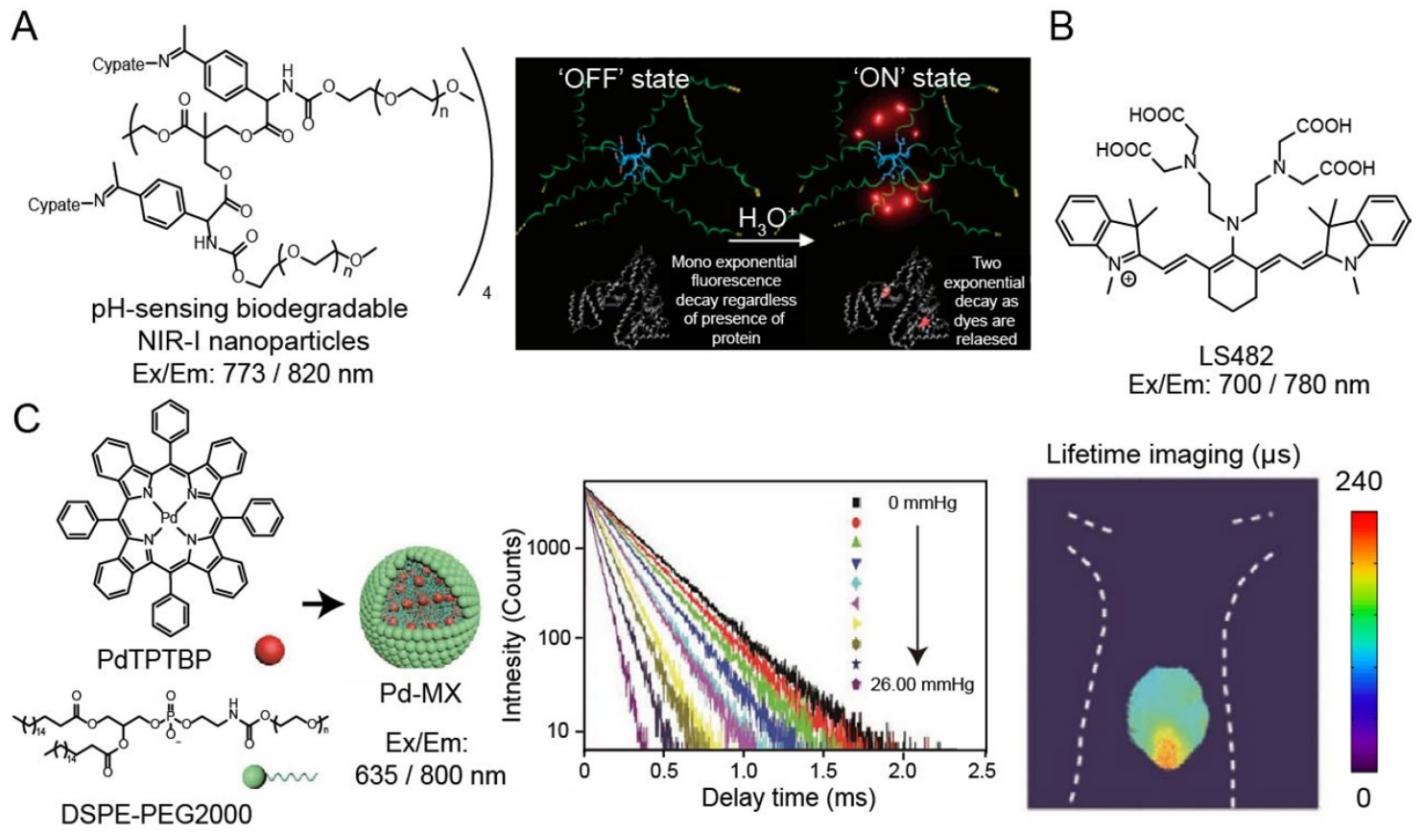
(A) Chemical structure of pH-sensing biodegradable NIR-I nanoparticles (ex/em: 773/820 nm) (left). The mechanism of pH sensing nanoparticles, “OFF: state: fluorescence lifetime of the nanoprobe is silenced in the neutral pH condition; “ON” state: fluorescence lifetime is increased when it binds to proteins in the acidic pH condition(right). Adapted with permission from 88, copyright 2008. (B) Chemical structure of pH probe LS482 (ex/em: 700/780 nm). (C) Illustration of synthesis of nanoparticles Pd-MX (left). Luminescence lifetime decay profile of the Pd-MX at different oxygen levels (middle). Luminescence imaging of liver tumors at 12 h after injection of Pd-MX (ex/em: 635/800 nm) (right). Adapted with permission from 90, copyright 2020.

Many oxygen-containing or nitrogen-containing organic dyes have protonated and deprotonated forms, leading to the variation in their luminescence intensity or emission wavelength at different pH values. The luminescence lifetime would also change along with the molecular luminescence states during the protonation or deprotonation processes. Thus, by measuring pH-induced changes in luminescence lifetime, the mapping of pH in cells and tissues can be obtained. Almutairi and coworkers have developed pH-sensing biodegradable NIR-I nanoparticles (ex/em: 773/820 nm) for luminescence intensity and lifetime imaging **(Figure 4A)** 76. Under neutral and alkaline environments, the NIR-I dye in the nanoparticles exists in the form of aggregates with weak luminescence and short lifetime. Under the acidic condition, nanoparticles would be broken and the released NIR-I dye bind to bovine serum albumin (BSA), resulting in increased luminescence intensity and lifetime (from 0.36 to 0.98 ns). Berezin and coworkers have also reported NIR-I pH-sensing luminescence lifetime molecular probe, LS482 (ex/em: 700/780 nm), with pKa ~5.5 for biological application **(Figure 4B)** 88. LS482 has shown excellent pH sensitivity lifetime change with two different molecular forms, acidic (∼1.16 ns) and basic forms (∼1.4 ns). Then, when the LS482 probe is administrated subcutaneously, the luminescence lifetimes signal exhibited robust values and are not influenced by tissue penetration. These results demonstrated the feasibility of pH sensing nanoparticles for luminescence imaging in animals.

Oxygen is an efficient luminescence quencher for almost all fluorophores due to collisional quenching. Strong quenching by oxygen is valuable for probes according to the decreased intensity or lifetime. Therefore, luminescence lifetime fluorophores, such as phosphorescent metal-ligand complexes (MLCs), are suitable for monitor tissue oxygenation. For example, Schreml and coworkers reported a palladium complex (ex/em: 444/797 nm) for imaging oxygen distribution and tissue hypoxia 89. Recently, Liu and coworkers have been developed an oxygen sensitive nanoparticle, Pd-MX (ex/em: 635/800 nm), containing palladium complex for quantitative mapping the distribution of oxygen in hypoxia hepatic tissue and hepatic tumor lesion in mouse *via* phosphorescence lifetime imaging **(Figure 4C)** 90. Additionally, researchers have also developed luminescence lifetime probes for detecting specific molecules, such as hydrogen peroxide and nitric oxide. Lifetime imaging of cells or tissues for these species is still evolving, and the development of molecular probes will help understand the physiological changes of related analytes.

### Luminescence lifetime imaging in NIR-II window

Compared with NIR-I region, photons in NIR-II region have deeper tissue penetration due to the reduced tissue scattering and absorbance 59,98. Thus, luminescence lifetime imaging in NIR-II region has more advantages in large animal models. Up to now, only rare-earth doped nanoparticles (RENPs) are used as contrast agents for NIR-II luminescence lifetime imaging due to the long and adjustable lifetime from microseconds to milliseconds. Similar to the classification criteria of NIR-I, luminescence lifetime imaging in NIR-II region can also be classified as non-activatable and activatable luminescence lifetime imaging.

#### Non-activatable luminescence lifetime imaging in NIR-II window

Due to tissue absorption and scattering of excitation and emission light, inhomogeneous signal attenuation occurs inevitably during luminescence bioimaging, which is the main problem for quantitative detection during multiplexed imaging. Luminescence lifetime that does not change with tissue penetration depth and probe concentration holds great value. Therefore, luminescence lifetime imaging is suitable for multiplexed imaging to observe the distribution of probes with different lifetimes in organ and tissue of interest. Zhang and coworkers have reported a general method based on controlled energy relay for RENPs to regulate their NIR-II luminescence lifetimes. Adjustable lifetimes can be achieved universally in NIR-II region by selecting various emitting ions, including Nd^3+^ at 1060 nm, Ho^3+^ at 1155 nm, Pr^3+^ at 1289 nm, Tm^3+^ at 1475 nm and Er^3+^ at 1525 nm 61. For example, Er^3+^-doped RENPs has a wide range of luminescence lifetime from 1.25 to 7.21 ms, which can be accommodate 11 distinct lifetime identities. Three kinds of Er^3+^-doped RENPs with different lifetimes were conjugated with three different antibodies to target estrogen receptor (ER), progesterone receptor (PR) and human epidermal growth factor receptor-2 (HER2) on breast cancer cells, which successfully achieved multiplexed imaging to distinguish different subtypes of breast tumor. These results demonstrated that multiplexed lifetime imaging could be used as a minimally invasive approach for disease diagnosis. Unfortunately, the reported RENPs have low quantum yields (0.009-0.235%), which leads to long data acquisition time and cannot realize rapid lifetime imaging.

#### Activatable luminescence lifetime imaging in NIR-II window

**Figure 5:**
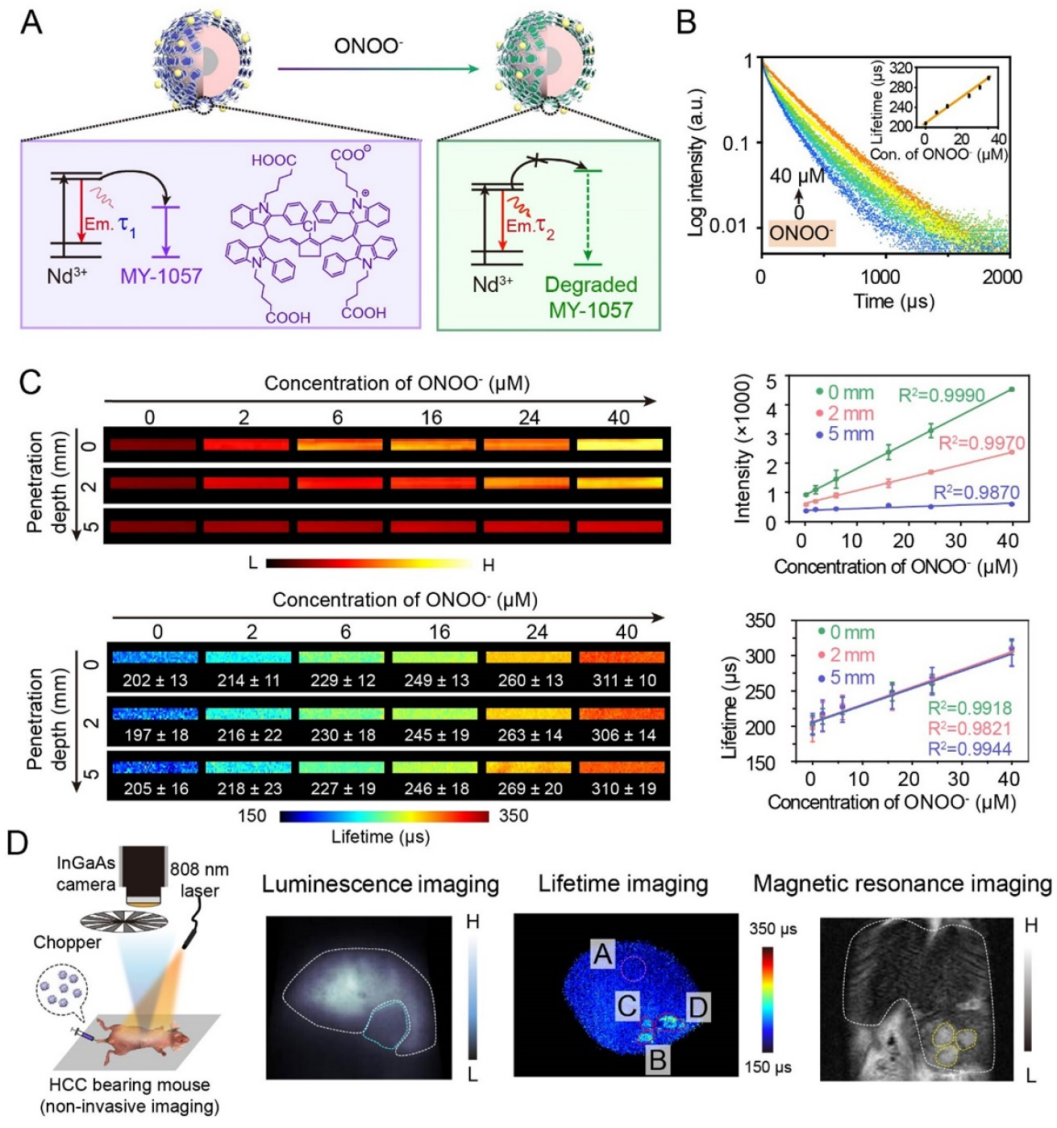
(A) Illustration of luminescence lifetime FRET sensor DSNP@MY-1057-GPC-3 for ONOO^-^ and hepatocellular carcinoma (HCC) detection in NIR-II region. (B) Luminescence lifetime decay profile of sensor at different concentration of ONOO^-^. (C) Luminescence intensity and lifetime imaging of sensor at different penetration depths (1% Intralipid) with increased ONOO^-^ concentration. (D) Experimental setup used to for NIR-II lifetime imaging (right side). NIR-II luminescence intensity imaging, luminescence lifetime imaging and magnetic resonance imaging (MRI) of HCC tumor. ROI A: healthy hepatic tissue; B-D: tumor lesions identified by lifetime imaging. Adapted with permission from 54, copyright 2020.

Thanks to deep tissue penetration of NIR-II imaging and reliability of lifetime imaging, activatable NIR-II lifetime imaging has great potential in biological detection. For example, an inorganic-organic hybrid probe has successfully achieved a corresponding lifetime change to detect biomolecules. Zhang and coworkers have reported NIR-II luminescence lifetime FRET sensor DSNP@MY-1057-GPC-3 for hepatocellular carcinoma (HCC) detection and quantitative ONOO^-^ sensing (**Figure 5A**) 54. Nd^3+^-doped RENPs with 1060 nm emission acted as FRET donor, and NIR-II absorption dye MY-1057 was chosen as FRET acceptor. Upon addition of ONOO^-^, a kind of reactive nitrogen species (RNS) in tumor, MY-1057 would be gradually decomposed, leading to lifetime recovery from 203 ± 2 to 298 ± 2 μs (**Figure 5B**). Compared with intensity-based imaging, luminescence lifetime-based detection was not influenced by tissue penetration depth, leading to a high reliablilty during quantitative *in vivo* ONOO^-^ detection (**Figure 5C**). These results indicated that lifetime imaging can quantitatively detect analytes under unknown tissue penetration depth. Due to excellent characteristics of lifetime imaging, multi-HCC lesions with lifetimes ranging from 215 ± 27 to 249 ± 43 μs were easily distinguished from normal tissues which had a lifetime of 205 ± 7 µs, while tumor lesions could not be identified by intensity-based imaging. Meanwhile, lifetime imaging, MRI and dissected imaging results were consistent, indicating the accuracy and reliability of lifetime imaging (**Figure 5D**). Due to the high stability and reliability, luminescence lifetime imaging is a promising and powerful imaging modality to provide qualitative and quantitative analysis ability for *in vivo* biosensing and bioimaging.

## Summary and Outlook

Luminescence intensity imaging will continue to be widely used in conventional bio-imaging and bio-sensing. Yet, luminescence lifetime imaging is a new technology, which has been rapidly developing in recent years. As the intrinsic property of luminescent contrast agents, lifetime imaging can provide more information that may not be obtained from intensity-based imaging. The independence of luminescence lifetime on the materials concentration, intensity and tissue penetration depth overcomes spectral overlap and background interference in wavelength or intensity dependent multiplexed imaging and detection. Therefore, lifetime imaging could not only perform qualitative, but also achieve quantitative analysis. Recently, the trend towards deeper tissue imaging with NIR light, especially NIR-II, has received extensive attention. Wide-field NIR-II luminescence lifetime imaging has been used for *in vivo* multiplexed imaging and bio-sensing, which provides a good basis for subsequent clinical applications.

Despite some progress, there are still many challenges to be addressed to further promote bioimaging and biosensing in this promising field. (1) The number and types of materials required for luminescence lifetime imaging are relatively small. (2) The design concept of luminescence lifetime activatable materials is relatively simple. (3) There are few reports on the detection of biomarkers or biomolecules by lifetime imaging. (4) Luminescence lifetime imaging instrumentation is expensive, and lifetime data acquisition is generally slow. In order to promote the development of luminescence lifetime imaging, several directions needed to be considered in future studies:

### Developing more luminescence lifetime probes with high stability and quantum yields

At present, only a small number and a few types of materials used for NIR luminescence lifetime imaging have been reported, including a few molecular dyes, transition metal-based complexes, and RENPs. In the next step, researchers may try to use other types of materials, such as QDs, SWCNTs, SPN etc. for lifetime imaging. The current materials for NIR activatable luminescence lifetime imaging mainly contain activatable molecules, transition metal-based complexes, and FRET nano-composites. As for NIR activatable luminescence lifetime materials based on FRET system, RENPs with long lifetime from μs to ms are suitable as donors. And the reported NIR activatable acceptors of FRET nano-composites are organic molecules. However, difficulties in designing and synthesizing FRET acceptors hinder the construction of FRET nano-composites, especially in NIR-II region. To solve this problem, researcher could develop more types of acceptors of FRET nano-composites. The performance of the lifetime probes, including high quantum yields and high stability (chemical stability and photostability), are the key factors for luminescence lifetime imaging. Higher quantum yield brings faster imaging speeds and less administration dosage. Higher stability can avoid false signal results. Unfortunately, most of the reported NIR lifetime probes have low quantum yields and poor stability. As for organic dyes, rational design strategies, including changing terminal groups, introduction rigid plane chemical structure can increase the stability and improve the quantum yields. As for RENPs, adjusting the type of doped ions and designing new structures also can achieve above goal.

### Developing more generic lifetime activatable design strategy

Activatable materials based on changes in luminescence intensity have been widely used for detecting or imaging specific biomarkers or physiological processes in living system. There are many reasons for the luminescence intensity change of the material, such as the change of the molecular aggregation state, and the energy transfer between luminescence materials and quenchers. This process generally leads to the change of the lifetime. Therefore, the traditional idea of designing luminescence intensity changes, such as FRET, intramolecular charge transfer (ICT), photoinduced electron transfer (PET), can be used to design activatable lifetime probes. Meanwhile, through these design strategies, more lifetime activatable probes can be developed to detect more biomolecules, such as enzyme, metal ions. In addition, another thing to note is that the variation range of the activatable lifetime of probe should be wide enough to provide high sensitivity and accuracy during detection.

### Activatable luminescence lifetime probes with multiplexed activation

The occurrence and development of diseases are generally accompanied by changes in multiple physiological indicators. Compared with current single-parameter activatable lifetime probes, lifetime probes which can simultaneously detect the changes of multiple physiological indexes, will significantly improve the accuracy of disease detection. This will be one of the future directions of activatable luminescence lifetime imaging.

### Combination of luminescence lifetime and theranostics

At present, a large amount of basic research works focused on simple luminescence lifetime without further consideration of therapy, which wastes the therapeutic potential of probes. By exploring the therapeutic potential of the lifetime probe itself, it can realize the diagnosis and treatment of diseases or observe the process of physiological changes under the guidance of luminescence lifetime imaging. Different types of lifetime probes have different optical and chemical functions. It is a good choice to further explore its diagnostic and therapeutic potential with minimal side effects and maximum therapeutic effects. In addition, the integration of diagnosis and therapy can also be achieved through simple loading or conjugated with drug.

### Developing luminescence lifetime imaging instrument

Progress in luminescence lifetime platforms strongly depends on imaging equipment for multiplexed imaging or quantitative detection. However, lifetime imaging instrumentation is more costly than intensity or wavelength measurements. In addition, lifetime data acquisition speed is generally slower than intensity imaging. This is mainly because the fluorescence lifetime of each pixel on the detector requires more photons to be collected and accurately calculated. This makes it impossible to monitor and image rapidly occurring biological processes. So far, the commonly used luminescence lifetime imaging system is based on a data acquisition card and an optical chopper to control time synchronization. The rotation speed of the optical chopper and the response time of the transistor-transistor logic modulated laser are the factors affecting the lifetime imaging speed. Meanwhile, introducing more than two pieces of achromatic lens into imaging system can cause the efficiency decrease of fluorescence collection. Therefore, the ideal imaging instrument should have short data acquisition and analysis time, high temporal-spatial resolution for both cellular and deep tissue imaging. In order to achieve the above goals, equipment modification and software upgrade are the main concerns. Developing more advanced algorithms and imaging systems, or using advanced computers can speed up data acquisition and processing.

### Clinical translation of luminescence lifetime imaging

Excellent probes with good biocompatibility, high quantum yields and stability, inexpensive equipment, and software upgrades are essential prerequisites for clinical translation. Lifetime imaging has almost no background interference, which is of great significance for clinical applications. First, the lifetime kits can be used to detect *in vitro* samples, such as tissues, blood, saliva, urine, feces, etc. Lifetime probes kits which can simultaneously detect multiple markers for a disease, can significantly improve the accuracy of disease detection. The results of the life test can be used as a supplement to the conclusions drawn by current clinical testing methods. Then, with the development of science and technology, lifetime-based imaging, and diagnostics, have been achieved at small animals' level, such as mice. Large animal measurement, even human-level measurement may also be realized if researchers could overcome the above-mentioned issues including probe and equipment development. For* in vivo* clinical applications, lifetime imaging can be used to detect some shallow tissue like skin diseases. Meanwhile, the development of lifetime endoscope imaging for deep tissue is also the direction of future development.

## Figures and Tables

**Table 1:** Summary of NIR-I and NIR-II luminescence lifetime materials

	Materials	Ex./Em. (nm)	Lifetime	Designed mechanism	Imaging applications	Ref.
NIR-I non-activatable materials	cypate and DTTCI	773 / 840	0.5 -1.1 ns	/	Multiple imaging	82
	Yb-doped RENPs	808 / 980	0.7 -1.3 ms	/	Multiple imaging	83
	Tm-doped RENPs	980 / 808	78 - 2157 μs	/	Multiple imaging	84
NIR-I activatable materials	MMPSense750 FAST	749 / 775	0.63 - 0.76 ns	Matrix metalloproteinase activatable FRET probe	Imaging of breast cancer	85
	PGC-800	778 / 794	0.29 ± 0.01 - 0.47 ± 0.01 ns.	cathepsin B activatable FRET probe	Imaging of mouse infarcted myocardium	86
	CdTeSe/ZnS QDs and LS662	488 / 750	29 ns at pH >7 12 ns at pH <5	pH activatable FRET probe	/	74
	Tm-doped RENPs and IR-820	785 / 800	200 - 600 µs	ClO^-^ activatable FRET probe	Imaging of arthritis in mice	87
	Cypate and aliphatic polyester dendrimer nanocomposites	773 / 820	0.36 - 0.98 ns	pH-activatable dye aggregation state change	/	76
	LS482	700 / 780	1.16 -1.4 ns	pH-induced dye protonated and deprotonated form change	/	88
	Palladium complex	444 / 797	50 - 300 µs	Oxygen-induced NPs	Imaging of oxygen distribution and tissue hypoxia	89
	Pd-MX	635 / 800	75 - 270 µs	Oxygen-induced NPs	Imaging of tumor lesion in mice liver	90
NIR-II non-activatable materials	Nd, Ho, Pr, Tm and Er-doped RENPs	808 / 1060 (Nd), 1155 (Ho), 1289 (Pr), 1475 (Tm),1525 (Er)	1.25- 7.21 ms	/	Multiple imaging	61
NIR-II activatable materials	DSNP@MY-1057-GPC-3	808 / 1060	203 ± 2 - 298 ± 2 μs	ONOO^-^- activatable FRET probe	Imaging of hepatocellular carcinoma	54

Ex.: Excitation wavelength; Em.: Emission wavelength.

## Abbreviations

BSA: bovine serum albumin
DTTCI: 3,3-diethylthiatricarbocyanine iodine
ER: estrogen receptor
FRET: Förster resonance energy transfer
HER2: human epidermal growth factor receptor-2
HCC: hepatocellular carcinoma
ICT: intramolecular charge transfer
MMP: matrix metalloproteinase
MRI: magnetic resonance imaging
MLCs: metal-ligand complexes
NIR: near-infrared
NIR-II: second near-infrared window
PR: progesterone receptor
QDs: quantum dots
SWCNTs: single-walled carbon nanotubes
SPN: semiconducting polymer nanomaterials
SWIR: shortwave infrared region
TCSPC: time correlated single photon counting
PET: photoinduced electron transfer
RENPs: rare-earth doped nanoparticles
ROS: reactive Oxygen Species
RNS: reactive nitrogen species
